# Outbreak of Cyclosporiasis Among Patrons of a Mexican-Style Restaurant — Limestone County, Alabama, May–June 2023

**DOI:** 10.15585/mmwr.mm7413a1

**Published:** 2025-04-17

**Authors:** Justine Goetzman, Adyneshia Carter, Ana Oliveira, L. Amanda Ingram

**Affiliations:** 1 Alabama Department of Public Health.

**Summary**

**What is already known about this topic?**

Cyclosporiasis is an intestinal illness caused by the parasite *Cyclospora cayetanensis*. In the United States, cyclosporiasis outbreaks are commonly associated with fresh, imported produce.

**What is added by this report?**

In June 2023, a total of 47 cases of cyclosporiasis were associated with consumption of food from a Mexican-style restaurant in Alabama. Analysis of case-control data identified cilantro as the likely food source. Collaboration among multiple states and their respective agencies enabled successful traceback of cilantro to a source in Mexico.

**What are the implications for public health practice?**

Cilantro imported from Mexico remains a food source of concern for cyclosporiasis. Distribution of potentially contaminated products via improper supply chain channels remains a public health challenge.

## Abstract

In early June 2023, the Alabama Department of Public Health identified five laboratory-confirmed cyclosporiasis case reports with a common patient exposure of eating at one Mexican-style restaurant. Common signs of cyclosporiasis include diarrhea, abdominal cramps, nausea, and loss of appetite. Although most illnesses are self-limited, antibiotic treatment can prevent relapsing illness. Onset of illness for the initial five patients occurred during May 26–30. An outbreak investigation was initiated on June 7. Routine case investigations and case finding through the restaurant’s food delivery service contact list identified 42 additional cases. Multivariable analysis of case-control study data revealed that illness was associated with consumption of cilantro (odds ratio = 40.9; 95% CI = 6.4–808.6). The cilantro was traced back to a Texas firm with no identified food manufacturing license that sourced the product from Mexico. The outbreak and its investigation demonstrate the ongoing need for regulatory controls of produce suppliers, documentation and review of business licenses, and increasing public awareness of food safety and outbreaks. Distribution of potentially contaminated products via improper supply chain channels remains a public health challenge. Avoiding infection in the United States involves preventing contaminated produce from reaching local retailers and consumers.

## Investigation and Results

### Epidemiologic Investigation

On June 7, 2023, the Alabama Department of Public Health (ADPH) opened an outbreak investigation after reviewing five reports of laboratory-confirmed cases of cyclosporiasis, an intestinal illness caused by the parasite *Cyclospora cayetanensis.* The case reports identified one Mexican-style restaurant as a common exposure. Onset of illness for all patients was May 26–30. Alabama’s Cyclosporiasis Investigation Form was used to interview the five persons in whom the initial confirmed cases were identified. An outbreak-specific questionnaire was created based on the menu provided by the restaurant and was distributed to patrons who ate at the restaurant May 20–June 6. Routine case investigations and case finding through the restaurant’s food delivery service contact list identified 42 additional cases. ADPH’s Institutional Review Board Primary Review Team determined that this study was exempt from the Federal Policy for the Protection of Human Research Subjects.

**FIGURE 1:**
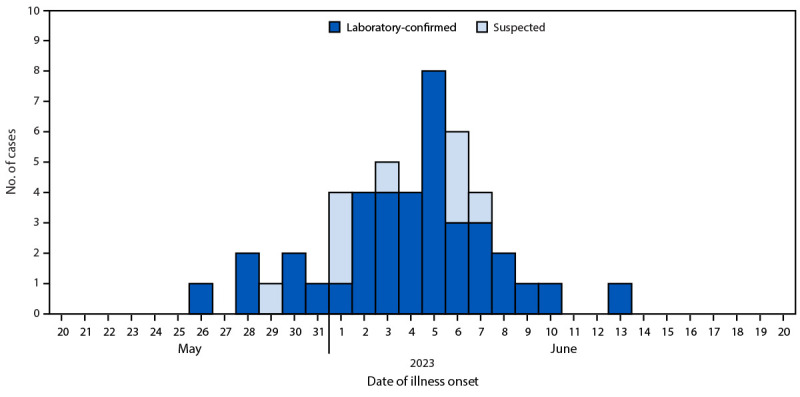
Laboratory-confirmed and suspected cases of cyclosporiasis associated with a Mexican-style restaurant, by case classification and date of illness onset (N = 47) — Limestone County, Alabama, May–June 2023

**Case-control study.** Case-patients (47) were persons who ate food from the restaurant during May 20–June 6 and developed diarrhea 2–14 days after their meal date. Thirty-eight cases (81%) with evidence of *Cyclospora cayetanensis* infection by polymerase chain reaction were considered confirmed; nine cases (19%) that lacked laboratory evidence were considered suspected. The control group (17) included persons who ate food from the restaurant May 20–June 6 and did not become ill (Figure 1). The median (IQR) incubation period was 7 (2–14) days. Most patients with cases of cyclosporiasis were female (32; 68%), White (47; 100%), and non-Hispanic (47; 100%); characteristics of control group subjects were similar: female (10; 59%), White (16; 94%), and non-Hispanic (17; 100%).

Case-control data were analyzed in a three-step analysis using Firth’s penalized likelihood logistic regression to minimize estimate bias caused by small sample sizes, rare events, and complete independence of patients infected with cyclosporiasis and control subjects, followed by stepwise variable selection with backward elimination. In step one, univariate odds ratios (ORs) and 95% CIs were calculated for each menu item. The only food item significantly associated with illness (p-value <0.05) in univariate analysis was salsa roja (OR = 20.5; 95% CI = 3.9–145.0). In step two, ORs and 95% CIs were calculated for each fresh produce ingredient. Four ingredients were significantly associated with illness on univariate analyses: cilantro (OR = 27.7), jalapeños (OR = 27.7), onions (OR = 22.2), and tomatoes (OR = 8.0) (Table). When these four ingredients were included in a multivariable analysis using Firth’s penalized likelihood regression, ORs for cilantro and jalapeños were elevated but not statistically significant (both 65.8; p-value = 0.161). In step three involving backward stepwise logistic regression only cilantro remained significantly associated with illness (OR = 40.9; 95% CI = 6.4–808.6) in a model where the four ingredients were included. The model was run multiple times with jalapeños listed last in the order of elimination and then cilantro listed last in the order of elimination. Data analysis was conducted using SAS software (version 9.4; SAS Institute).

**TABLE:** Three-step case-control analysis of food items consumed by patrons of a Mexican-style restaurant — Limestone County, Alabama, May 20–June 6, 2023

Step 1. Univariate analysis of menu items				
**Menu item**	**Case-patients (47)**	**Control subjects (17)**	OR* (95% CI)	**p-value**
Salsa roja	42	4	20.5 (3.9–145.0)	0.001
Pico de gallo^†^	10	2	2.2 (0.4–12.6)	0.36
Taco	2	5	0.5 (0.1–3.2)	0.48
Burrito	7	1	2.1 (0.2–18.8)	0.52
Pollo con arroz	5	3	0.6 (0.04–8.1)	0.67
**Step 2. Ingredient-level analysis**				
**Ingredient**				
Cilantro	46	9	27.7 (5.3–283.9)	0.001
Jalapeño	46	9	27.7 (5.3–283.9)	0.001
Onion	46	10	22.2 (4.2–227.1)	0.002
Tomato	45	12	8.0 (1.7–49.2)	0.018
Bell pepper	6	1	1.7 (0.3–17.5)	0.59
Lime juice	15	4	1.4 (0.4–5.4)	0.58
Mushroom	1	0	1.1 (0.1–168.0)	0.96
Iceberg lettuce	18	7	0.9 (0.3–2.7)	0.82
Romaine lettuce	6	3	0.7 (0.2–3.0)	0.57
Avocado	11	5	0.7 (0.2–2.5)	0.60
Garlic	2	1	0.6 (0.1–7.0)	0.68
Chile	0	1	0.1 (<0.001–2.3)	0.36
Poblano	0	1	0.1 (<0.001–2.3)	0.36
**Step 3. Multivariable analysis of cilantro, jalapeños, onions, and tomatoes using backward stepwise logistic regression**				
Cilantro			40.9 (6.4–808.6)	0.001

**Abbreviation:** OR = odds ratio.

* ORs were estimated using Firth’s penalized likelihood regression method to minimize the analytical bias caused by small samples, rare events, and complete separation of case-patients with cyclosporiasis and control subjects.

^†^ Mexican salsa made with tomatoes, onions, jalapeños, cilantro, and lime juice.

### Environmental Health Investigation

On June 9, the ADPH Bureau of Environmental Services conducted an environmental assessment of the restaurant. This assessment included a food flow (*1*) for ingredients or menu items that contain fresh, uncooked produce: salsa roja, taco salad, queso, guacamole, chicken soup, pico de gallo (Mexican salsa made with tomatoes, onions, jalapeños, cilantro, and lime juice), salsa verde, and cilantro; these items are commonly associated sources for cyclosporiasis outbreaks. The environmental assessment revealed no substantial findings. Because all ingredients used during the period when patients ate at the restaurant had been discarded before the environmental assessment, no laboratory testing of restaurant ingredients was performed. Invoices obtained from the restaurant indicated that fresh produce was received during May 25–June 5 from a distributor in Georgia. The invoices were shared with Georgia Department of Public Health and Georgia Department of Agriculture on July 10.

**Laboratory investigation.** Stool specimens were collected from patients with suspected cyclosporiasis. Some specimens were collected by health care providers and then forwarded to the ADPH Bureau of Clinical Laboratories (BCL), whereas other specimens were collected by ADPH. After specimens were received by BCL, they were forwarded to CDC for genotyping. Among the 38 laboratory-confirmed cyclosporiasis cases, 29 were successfully genotyped by CDC. Genotyped specimens were assigned to one of six temporal-genetic cluster codes. Of those genotyped specimens, 23 (79%) were assigned to 2023_012, two (7%) to 2023_015, and one (3%) each to 2023_002, 2023_024, 2023_041, and 2023_069 temporal-genetic cluster codes. A large percentage of specimens with the same temporal-genetic cluster code suggests specimens were genetically related and likely linked to the same source. 

## Public Health Response

**FIGURE 2:**
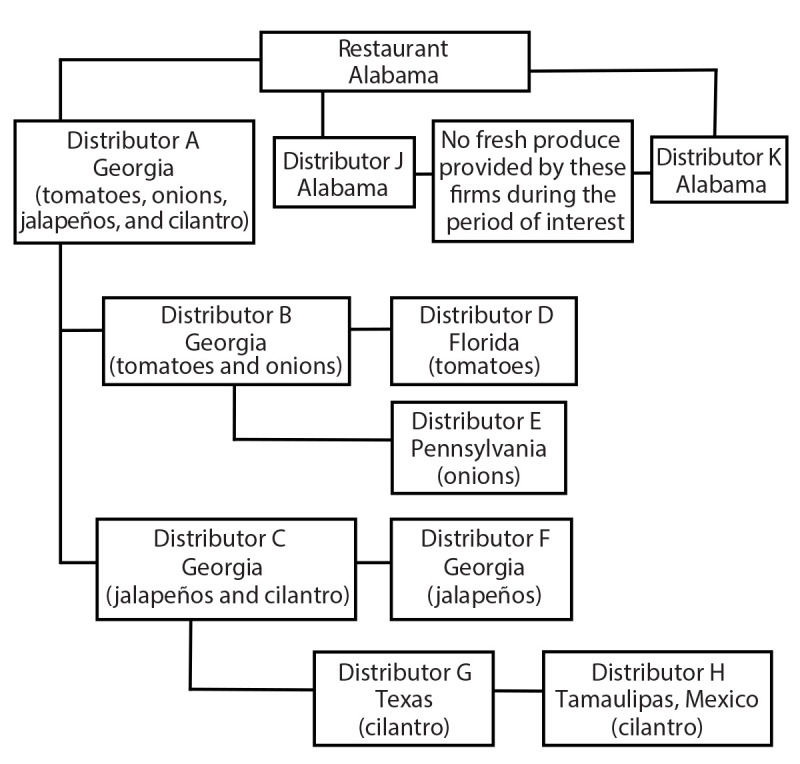
Traceback diagram of ingredients suspected in cyclosporiasis outbreak associated with a Mexican-style restaurant* — Limestone County, Alabama, June 2023 * The restaurant purchased produce from three different distributors, but only one distributor, distributor A in Georgia, provided produce during the time of interest for the outbreak. Distributor A supplied tomatoes, onions, jalapeños, and cilantro during the time of interest. Distributor A purchased the tomatoes and onions from distributor B in Georgia. Distributor B purchased the tomatoes from distributor D in Florida and purchased the onions from distributor E in Pennsylvania. Distributor A purchased the jalapeños and the cilantro from distributor C in Georgia. Distributor C purchased the jalapeños from distributor F in Georgia. Distributor C purchased the cilantro from distributor G in Texas. Distributor G purchased the cilantro from distributor H in Tamaulipas, Mexico.

ADPH issued a statewide Health Action Network alert to Alabama health providers on June 14 and a news release on June 15 to notify the public that the number of cyclosporiasis cases had increased since the beginning of June. ADPH also provided recommendations to the restaurant that included changing produce suppliers and increasing sanitation measures such as using high heat to clean utensils and other food preparation tools because *C. cayetanensis* is resistant to routine chemical disinfection or sanitizing methods and can be destroyed by high heat (*2*). Because the restaurant purchased produce from a supplier in Georgia, the Georgia Department of Agriculture’s Rapid Response Team (GA RRT) began traceback for cilantro, jalapeños, onions, and tomatoes provided to the restaurant by distributor A on July 10 (Figure 2). GA RRT reached out to its counterparts in Florida, Pennsylvania, and Texas upon discovering that distributor A obtained produce from distributors B and C, who reported acquiring produce from distributors in other states.

Because distributor A was a Georgia-based firm, GA RRT led the traceback investigation. Distributor A provided the requested invoices for the following suspected produce sources: cilantro, jalapeños, onions, and tomatoes. Distributor A purchased tomatoes and onions from distributor B. Tomatoes were supplied by a firm in Florida (distributor D) and onions by a firm in Pennsylvania (distributor E). Cilantro and jalapeños were purchased from distributor C (Georgia). Jalapeños were supplied by a firm in Georgia (distributor F), and cilantro was supplied by a Texas firm (distributor G).

GA RRT communicated its findings about distributor C to the Texas Department of Agriculture’s Rapid Response Team (TX RRT); a food manufacturing license for this firm could not be identified. Records obtained by TX RRT indicate that the cilantro was purchased from an import-export firm (distributor H) located in Tamaulipas, Mexico, and was held in cold storage in Texas before the sale to other firms across the United States. TX RRT could not determine where in Mexico the cilantro was grown on the basis of records provided by distributor C (Figure 2).

## Discussion

Epidemiologic, laboratory, environmental, and traceback evidence from this outbreak of cyclosporiasis among patrons of a Mexican-style restaurant linked the illness to contaminated cilantro imported from Mexico. Cilantro from Mexico has been identified as a food source for multiple cyclosporiasis infections during the past 3 decades (*3*). Collaboration among agencies, including state departments of health and agriculture and CDC, was essential to the successful traceback of the food source implicated by multivariable analysis. Genotyping data further supported the suspicion that cases from the restaurant were related.

*C*. *cayetanensis*, the parasite that causes cyclosporiasis, is resistant to routine chemical disinfection or sanitizing methods, limiting the ability for restaurant operators and food distributors to eliminate risk for contamination through effective sanitation practices (*3*). Because the restaurant’s environmental assessment report included no substantial findings, contamination likely occurred before arrival at the restaurant. ADPH recommended that the restaurant change suppliers for fresh produce to avoid the need for restaurant closure. The ADPH press release advised the public to thoroughly wash fresh produce before eating, cutting, or cooking (*4*). Because states do not have authority to conduct investigations across international borders, ADPH was unable to determine whether contamination occurred pre- or postharvest. Collaboration with partner organizations is essential to tracing ingredients back to their source and to identifying contributing factors and environmental antecedents at each step of the food supply chain. Through this investigation, a domestic distributor without an identified manufacturing license who sourced produce from an international supplier was discovered, presenting an opportunity for regulatory intervention and education to prevent the future sale and distribution of potentially contaminated product through improper supply channels.

### Limitations

The findings of this report are subject to at least three limitations. First, the wide CIs around the risk estimates indicate reduced statistical power to estimate precise risk associated with the implicated food ingredients, likely due to a small sample size and an imbalance favoring cases over controls (ratio = 2.8:1). Second, the backward stepwise regression procedures used can lead to biased estimates and be order-dependent, although, to reduce this bias, the stepwise model included all four ingredients from step two and was run with multiple orders of ingredients, with both cilantro and jalapeños in the last elimination step. Finally, testing cilantro from the grower or distributor to confirm the presence of *C. cayetanensis* in the ingredient as indicated by the epidemiologic analysis was not possible. 

### Implications for Public Health Practice

Because of the global nature of the U.S. food supply and the interconnectedness among growers, distributors, and consumers, rapid collaboration is essential for effective traceback investigations and implementation of control measures in response to foodborne illness outbreaks. These findings highlight the need for active disease surveillance by public health teams, regulatory oversight of food distributors, and focused educational initiatives for both distributors and restaurants to reduce future risk of distribution of contaminated products.
